# Engineering and functional characterization of a proton-driven β-lactam antibiotic translocation module for bionanotechnological applications

**DOI:** 10.1038/s41598-021-96298-4

**Published:** 2021-08-26

**Authors:** Mirko Stauffer, Zöhre Ucurum, Daniel Harder, Dimitrios Fotiadis

**Affiliations:** Institute of Biochemistry and Molecular Medicine, University of Bern, 3012 Bern, Switzerland

**Keywords:** Membrane proteins, Permeation and transport, Membrane structure and assembly, Structural biology

## Abstract

Novel approaches in synthetic biology focus on the bottom-up modular assembly of natural, modified natural or artificial components into molecular systems with functionalities not found in nature. A possible application for such techniques is the bioremediation of natural water sources contaminated with small organic molecules (e.g., drugs and pesticides). A simple molecular system to actively accumulate and degrade pollutants could be a bionanoreactor composed of a liposome or polymersome scaffold combined with energizing- (e.g., light-driven proton pump), transporting- (e.g., proton-driven transporter) and degrading modules (e.g., enzyme). This work focuses on the engineering of a transport module specific for β-lactam antibiotics. We previously solved the crystal structure of a bacterial peptide transporter, which allowed us to improve the affinity for certain β-lactam antibiotics using structure-based mutagenesis combined with a bacterial uptake assay. We were able to identify specific mutations, which enhanced the affinity of the transporter for antibiotics containing certain structural features. Screening of potential compounds allowed for the identification of a β-lactam antibiotic ligand with relatively high affinity. Transport of antibiotics was evaluated using a solid-supported membrane electrophysiology assay. In summary, we have engineered a proton-driven β-lactam antibiotic translocation module, contributing to the growing toolset for bionanotechnological applications.

## Introduction

**Figure 1:**
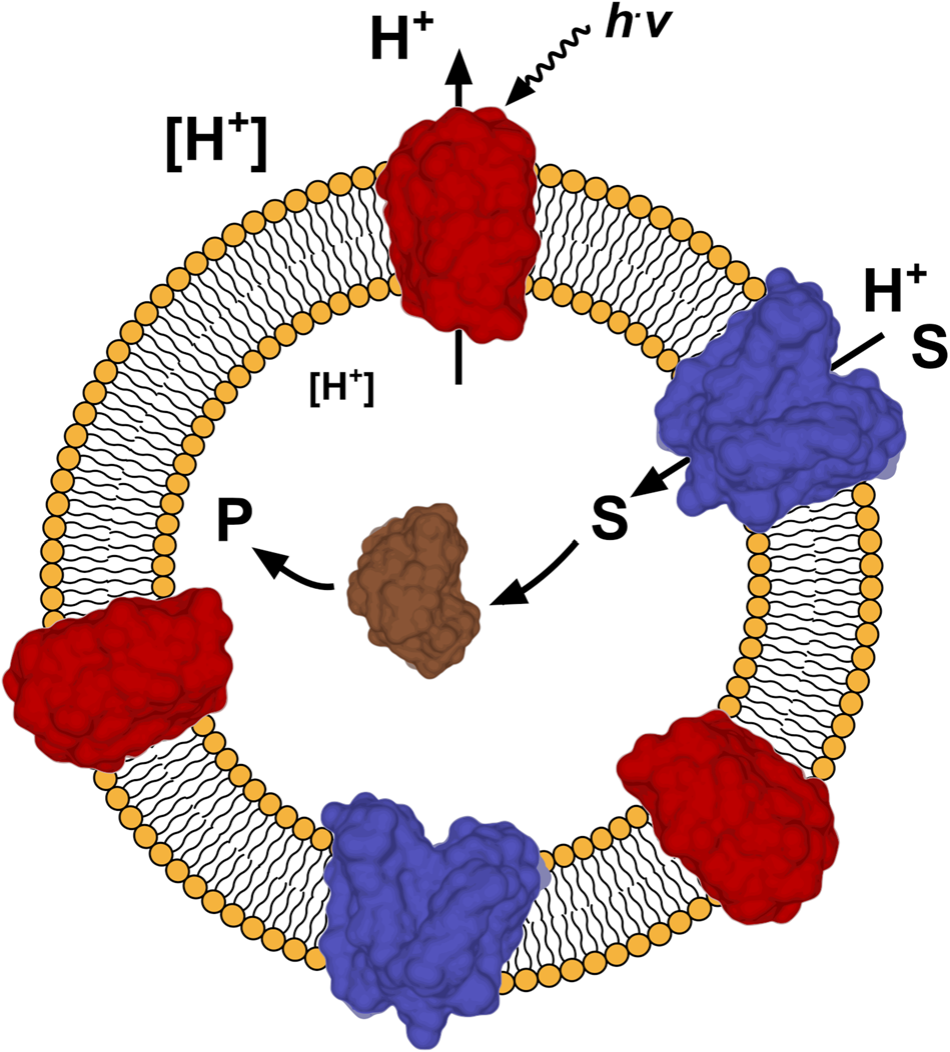
Schematic representation of a molecular system for the uptake and degradation of small organic molecules. A light-driven proton pump (red) is activated by light (*h*^.^*v*) generating a proton gradient (indicated by different sizes of [H^+^]) across a vesicular structure (e.g., liposome, in orange). A proton-driven transporter (in blue) accumulates the target substrate (S), e.g., a $\upbeta$-lactam antibiotic, inside the vesicle using the established proton gradient. Enzymes (in brown) entrapped inside the vesicle degrade the substrate to a non-active compound (P). Depicted modules are based on structures of proteorhodopsin (in red, PDB ID code: 2L6X), YePEPT (in blue, PDB ID code: 4W6V) and TEM1 β-lactamase (in brown, PDB ID code: 1BTL).

In synthetic biology, components and systems involved in biological processes are optimized or repurposed using engineering approaches to address specific challenges in a wide range of fields such as diagnostics, biotechnology and research^1,2^. This can be achieved by simplifying and/or manipulating existing biological systems for specific purposes (top-down approach)^1,3^. In contrast, bottom-up approaches combine natural, modified natural and artificial components with specific functions, called modules, to build completely new biological systems, which mimic natural processes or exhibit functionalities not found in nature^1,4,5^. One problem commonly addressed with synthetic biology principles^3,5^ is the growing threat of contamination of environments by small organic molecules such as drugs and pesticides, which cannot be removed by currently used mechanical or biological wastewater disposal techniques^6^﻿. Especially antibiotics are a growing body of concern, as they lead to a higher occurrence of antibiotic resistant genes in prokaryotic organisms^7,8^, which contributes to the formation of multiresistant pathogen strains. A simple molecular system to take up and degrade such pollutants could be a bionanoreactor (Fig. 1) made up of a scaffold module (e.g., lipids or block copolymers, Fig. 1, in orange), an energy-providing module (e.g., light-driven proton pumps, Fig. 1, in red), a transporting module (e.g., proton-driven membrane transporters for the target pollutant, Fig. 1, in blue), and a degrading module (e.g., enzymes or chemical catalysts, which are able to degrade the pollutants, Fig. 1, in brown)^4^. Potential pollutants for a proof-of-concept of such a molecular system represent β-lactam antibiotics. As mentioned before, such a system requires a transport protein to efficiently take up β-lactam antibiotics into bionanoreactors, preferably a proton-driven one to be able to energize it using a light-driven proton pump (Fig. 1).

A promising transporting module with the previously described requirements would be a peptide transporter from the proton-driven oligopeptide transporter (POT) family^9–12^ (also known as the peptide transporter family (PTR)^10,13^), which belong to the major facilitator superfamily (MFS)^14,15^. They are secondary active symporters^16,17^ and use the inwardly-directed electrochemical gradient to actively transport and accumulate their substrates across cellular membranes^11^. In human metabolism, the high-capacity, low-affinity peptide transporter PEPT1 (SLC15A1) is responsible for the uptake of dietary di- and tripeptides by epithelial cells in the small intestine, while the low-capacity, high-affinity peptide transporter PEPT2 (SLC15A2) reabsorbs di- and tripeptides in the kidney^18^. Beside those metabolic functions, both transporters and bacterial homologues were shown to also bind and transport a wide range of peptidomimetic drugs and prodrugs^19^ such as (β-lactam) antibiotics^20–25^ (e.g., cefadroxil, chloramphenicol), antivirals^24,26–28^ (e.g., valaciclovir, valganciclovir), protease inhibitors^24,29^ (e.g., bestatin) and Parkinson medications^24,26^ (e.g., l-dopa-l-Phe, L-dopa).

To be suitable for application in a bioremediation system, a high-affinity transporter like PEPT2 is needed to be able to take up the target molecule at low concentrations. In addition, components to be used in bionanotechnological applications, have to show good thermal stability^2^ and have to be producible in large quantities, i.e., in milligram amounts. Unfortunately, human proteins often show poor thermal stability and low yields when expressed in eukaryotic expression systems. Production using conventional bacterial expression systems is oftentimes not possible due to the lack of complex post-translational modifications needed for proper function of eukaryotic proteins^30^. Furthermore, reconstitution of human membrane proteins into liposomes and polymersomes is challenging, because of poor stability when solubilized in detergents and potential requirements to the membrane environment (i.e., specific phospholipid and sterol composition^31^). In contrast, bacterial homologues are more suitable for such applications, as they are easier to overexpress, more stable and less demanding in terms of lipid environment. We previously solved the ligand-free inward-facing crystal structure of the prokaryotic proton-driven peptide transporter YePEPT from *Yersinia enterocolitica* at 3 Å resolution^32^. Furthermore, we established a bacterial uptake assay to evaluate the specificity and affinity of YePEPT for different compounds by transport inhibition of the reporter radioligand [^3^H]Ala-Ala, as well as a solid-supported membrane (SSM) electrophysiology assay to assess transport of potential substrates.

This work focuses on the development of a molecular module from YePEPT, which is able to transport certain β-lactam antibiotics for application in a bioremediation system as described above (Fig. 1) and as a contribution for the growing toolset of modules, which can be used for molecular systems engineering. By combining structure-based mutagenesis with functional characterization, we were able to identify specific mutations, which enhance the affinity of YePEPT for certain β-lactam antibiotics into the low micromolar range. Using electrophysiology, we could demonstrate transport of identified compounds and therefore the suitability of the engineered YePEPT version as a β-lactam antibiotic translocation module.

## Results and discussion

### β-lactam antibiotic specificity determination of YePEPT^WT^

**Figure 2:**
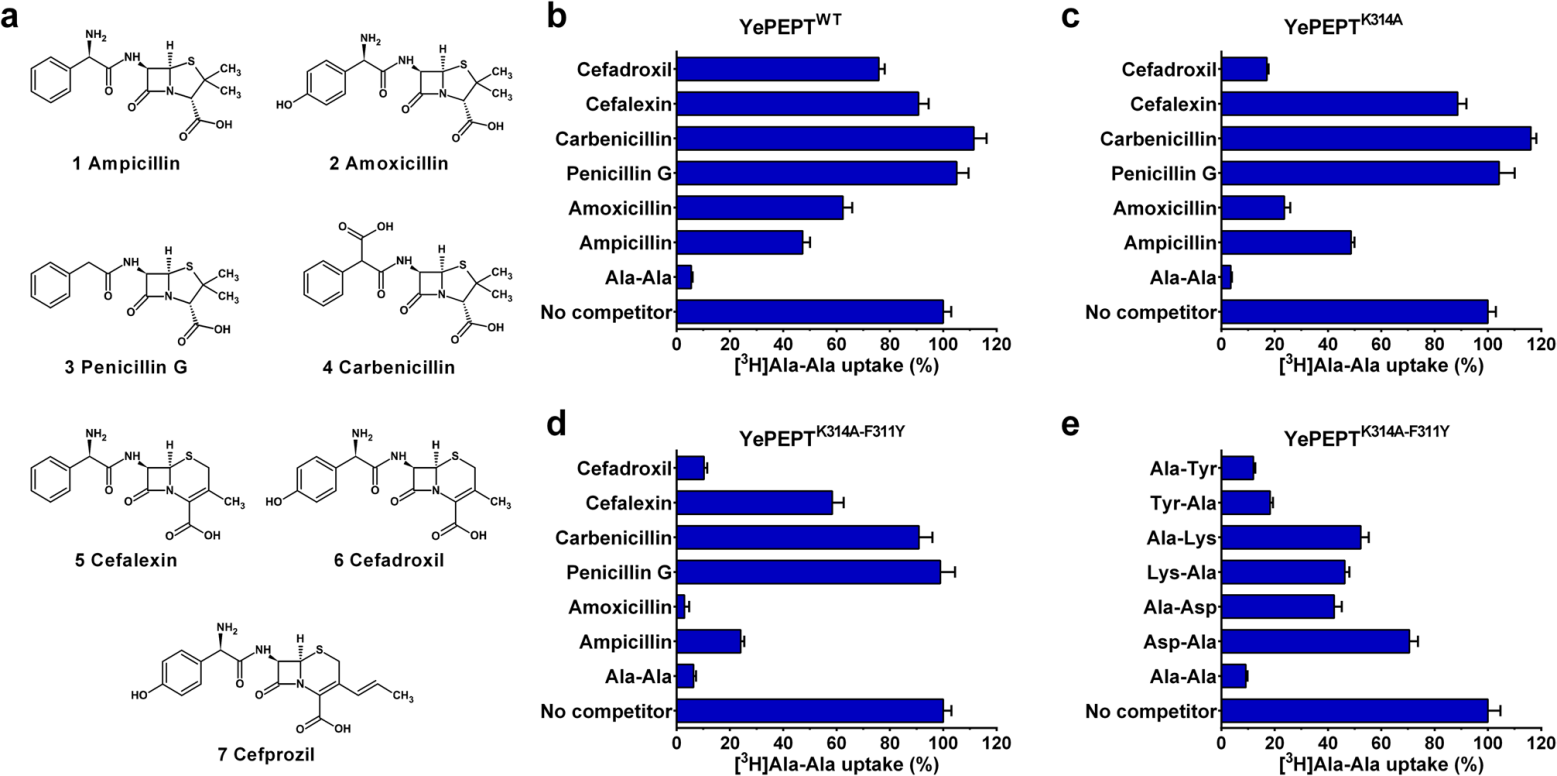
Specificity of YePEPT variants for β-lactam antibiotics and dipeptides. (**a**) Molecular structures of the screened β-lactam antibiotics. Specificity of (**b**) YePEPT^WT^, (**c**) YePEPT^K314A^ and (**d**) YePEPT^K314A-F311Y^ for selected penicillins and cephalosporins by competition assay (5 mM final concentration). **e** Specificity of YePEPT^K314A-F311Y^ for selected dipeptides by competition assay (2.5 mM final concentration). Bars in (**b**)–(**e**) represent vector-subtracted uptake of [^3^H]Ala-Ala normalized to the uninhibited signal ± SEM of at least three independent experiments, each at least in triplicate.

To determine the β-lactam antibiotic specificity of wild-type YePEPT (YePEPT^WT^), uptake inhibition of the reporter radioligand [^3^H]Ala-Ala by a set of six common and structurally diverse β-lactam antibiotics (Fig. 2a) at a concentration of 5 mM was evaluated in *Escherichia coli* cells overexpressing the transporter (Fig. 2b). This set consists of four penicillin- and two cephalosporin antibiotics. For the group of aminopenicillins (penicillins containing an α-amino group) the prototypical ampicillin (Fig. 2a 1) and its analogue amoxicillin (Fig. 2a 2), which contains a hydroxyphenyl- instead of a phenyl group, were selected. To evaluate the influence of the α-amino group, which corresponds to the N-terminal amino group of peptide substrates^33^, penicillin G (Fig. 2a 3) and carbenicillin (Fig. 2a 4) were included. In those compounds, the α-amino group is substituted with a hydrogen atom (penicillin G) or a carboxyl group (carbenicillin), respectively. All penicillins contain a penam ring structure consisting of a four-membered β-lactam ring and a five-membered thiazolidine ring fused together. Cephalosporins, in contrast, contain a cephem ring structure, in which the four-membered β-lactam ring is fused with a six membered thiazine ring. To evaluate the influence of those core-structures, cefalexin (Fig. 2a 5) and cefadroxil (Fig. 2a 6) were included, which are the cephalosporin analogues of ampicillin and amoxicillin, respectively. In addition to the selected antibiotics, a positive control without competitor and a negative control with unlabelled Ala-Ala as competitor were included in every screen. A time course analysis of [^3^H]Ala-Ala uptake by YePEPT^WT^ was conducted to ensure that all functional data is acquired in the linear regime (Supplementary Fig. S1).

The antibiotic inhibition profile of YePEPT^WT^ (Fig. 2b) showed an [^3^H]Ala-Ala uptake inhibition of around 50% for both aminopenicillins, with a slight preference for a phenyl- (i.e., ampicillin) over a hydroxyphenyl group (i.e., amoxicillin). Both non-aminopenicillins (i.e., penicillin G and carbenicillin) did not show any inhibition at all, indicating that the α-amino group is necessary for binding to the protein. The aminocephalosporins showed only a weak uptake inhibition, which points to a preference of a penam- over a cephem core structure. In contrast to the aminopenicillins, a slight preference for a hydroxyphenyl- (i.e., cefadroxil) over a phenyl group (i.e., cefalexin) could be observed.

### Structure-based mutagenesis of YePEPT^WT^ and β-lactam antibiotic specificity determination of YePEPT^K314A^

**Figure 3:**
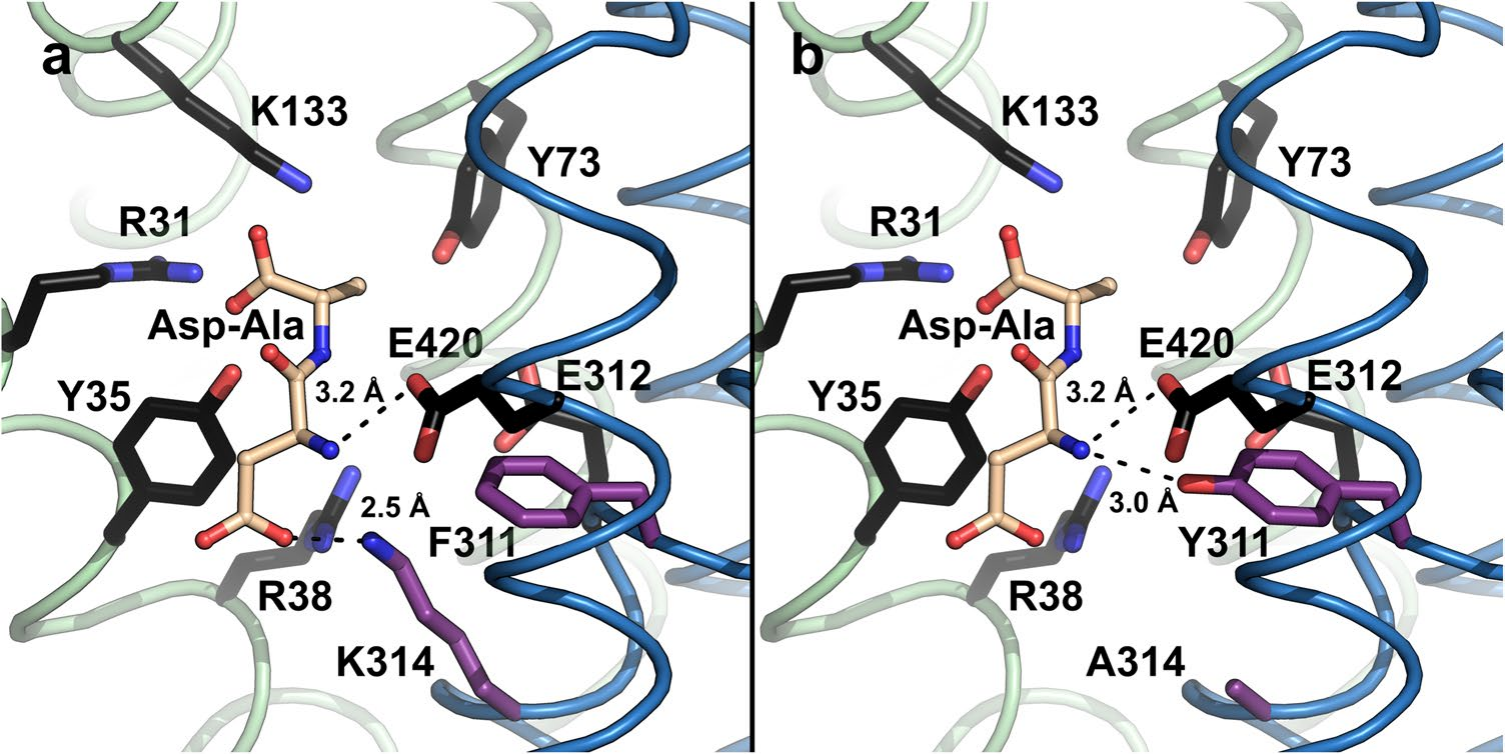
Model of the substrate binding site of YePEPT. (**a**) YePEPT^WT^ structure (PDB ID code: 4W6V^32^) with modelled bound Asp-Ala dipeptide. (**b**) YePEPT^K314A-F311Y^ structure with modelled bound Asp-Ala dipeptide. Distances between the nitrogen atom of K314 and the carboxyl group of the aspartate side chain of Asp-Ala (**a**), between the hydroxyl group of Y311 and the α-amino group of Asp-Ala (**b**), and between the carboxyl group of E420 and the α-amino group of Asp-Ala (**b**) are indicated. Conserved residues involved in peptide backbone interactions in POTs are coloured in black^34,35^. Non-conserved residues in the substrate binding pocket are coloured in purple. The N- and C-terminal bundles of the YePEPT^WT^ structure are coloured in green and blue, respectively. The Asp-Ala dipeptide was modelled into the binding pocket of YePEPT^WT^ (PDB ID code: 4W6V) by superposition with the Ala-Phe-bound structure of PepT_st_ in PyMol (The PyMol Molecular Graphics System, Schrödinger), followed by mutagenesis of the dipeptide as described^32^.

Most residues involved in interactions with the backbone of di- and tripeptides are highly conserved in the POT family and were shown to be involved in substrate binding and transport (Fig. 3a, in black)^34,35^. Therefore, these residues cannot be mutated to tune and improve the specificity and affinity of POTs towards β-lactam antibiotics. K314 (Fig. 3a, in purple), one of the few non-conserved residues in the substrate binding pocket of YePEPT^WT^, was previously shown to be responsible for the recognition of dipeptides containing amino acids with negatively charged side chains at the N-terminal position through ionic interactions (Fig. 3a, dashed line). When mutated to glutamate (K314E), the specificity was inverted towards a dipeptide with a positively charged amino acid side chain at the N-terminal position^32^. As all tested β-lactam antibiotics contain an uncharged group (i.e., a phenyl- or hydroxyphenyl-group) at the position corresponding to the N-terminal amino acid side chain of dipeptides, we expected a potential beneficial effect by removing the positively charged K314 in YePEPT^WT^. In addition, the removal of this lysine side chain opens up space in the binding pocket, which was also considered to be beneficial, as β-lactam antibiotics are larger than dipeptides. Time course analysis of [^3^H]Ala-Ala uptake by the mutant YePEPT^K314A^ demonstrated that the transport function is not impaired (Supplementary Fig. S1).

Compared to wild-type (Fig. 2b), the inhibition pattern for YePEPT^K314A^ (Fig. 2c) showed a strong increase in affinity for amoxicillin and cefadroxil, while ampicillin and cefalexin, as well as penicillin G and carbenicillin showed similar behaviours. We concluded that the K314A mutation specifically increases the specificity for hydroxyphenyl containing β-lactam antibiotics, which might be due to the additional space to accommodate the hydroxyl group and/or a new interaction with the protein (e.g., a hydrogen bond (H-bond) of the hydroxyphenyl group with the protein).

### Structure-based mutagenesis of YePEPT^K314A^ and β-lactam antibiotic specificity determination of YePEPT^K314A-F311Y^

To further increase the affinity of YePEPT^K314A^ for β-lactam antibiotics, we looked for other residues in the binding pocket, which can be mutated without impairing the transport function of YePEPT. A prominent non-conserved residue pointing towards the substrate binding site in YePEPT is F311 (Fig. 3a, in purple). It was previously proposed that F311 forms a cation-π interaction with K314 in YePEPT^WT^, thus stabilizing this positive charge in the substrate binding pocket in absence of bound negatively charged dipeptides^32^. When mutated to a tyrosine (F311Y) in silico (Fig. 3b, in purple), the oxygen atom of the additional hydroxyl group is within H-bond distance of the α-amino group of the previously modelled^32^ Asp-Ala dipeptide (Fig. 3b, dashed line). The α-amino group probably already forms an ionic interaction with E420, as the corresponding conserved residue in other POTs was shown to be involved in interactions with the α-amino group of bound dipeptides^36–38^. The distance of 3.2 Å from the carboxyl group of E420 in the structure of YePEPT^WT^ to the α-amino group of the modelled Asp-Ala dipeptide (Fig. 3b, dashed line), supports this assumption. The predicted additional interaction of Y311 with the dipeptide backbone was hypothesized to increase the specificity and affinity for dipeptides as well as for peptidomimetics containing an α-amino group (e.g., aminopenicillins and aminocephalosporins). Time course analysis of [^3^H]Ala-Ala uptake using YePEPT^K314A-F311Y^ expressing bacteria demonstrated that the transport function is retained in this mutant (Supplementary Fig. S1).

The antibiotic inhibition pattern of YePEPT^K314A-F311Y^ (Fig. 2d) showed an increased inhibition by all four β-lactam antibiotics containing an α-amino group (i.e., ampicillin, amoxicillin, cefalexin and cefadroxil) compared to YePEPT^K314A^, while there was still no inhibition by the non-aminopenicillins (i.e., penicillin G and carbenicillin). This behaviour fits with the hypothesis that the introduced tyrosine residue forms an additional H-bond with the substrate, most probably with the α-amino group of peptidomimetic substrates. To further test this hypothesis, the specificity of YePEPT^K314A-F311Y^ for selected dipeptides containing an acidic- (i.e., aspartic acid), a basic- (i.e., lysine) or an aromatic residue (i.e., tyrosine) at the N- or C-terminal position was determined at a concentration of 2.5 mM (Fig. 2e). The same experiment with YePEPT^WT^ showed previously, that only the tyrosine-containing dipeptides (i.e., Tyr-Ala and Ala-Tyr) as well Asp-Ala were able to inhibit [^3^H]Ala-Ala uptake^32^. In YePEPT^K314E^ the specificity changed from Asp-Ala to Lys-Ala while there was no change on inhibition by the tyrosine-containing dipeptides. This led to the conclusion that a positively or negatively charged side chain at position 314 plays a role in substrate recognition of dipeptides containing a charged residue at the N-terminal position. This hypothesis was strengthened by the fact that in YePEPT^K314A^ only the tyrosine-containing but none of the charged dipeptides were able to inhibit transport. In contrast, for YePEPT^K314A-F311Y^, all four charged dipeptides (i.e., Asp-Ala, Ala-Asp, Lys-Ala and Ala-Lys) showed a reduction of [^3^H]Ala-Ala uptake (~ 40–70% residual uptake). The aromatic dipeptides (i.e., Tyr-Ala and Ala-Tyr) showed a slightly stronger inhibition compared to YePEPT^K314A^ ^32^. These results support the hypothesis that the F311Y mutation leads to stronger interactions of YePEPT with ligands containing an α-amino group, independently of the side chains of the compound.

### Functional characterization of YePEPT^K314A-F311Y^ in ***E. coli*** cells

**Figure 4:**
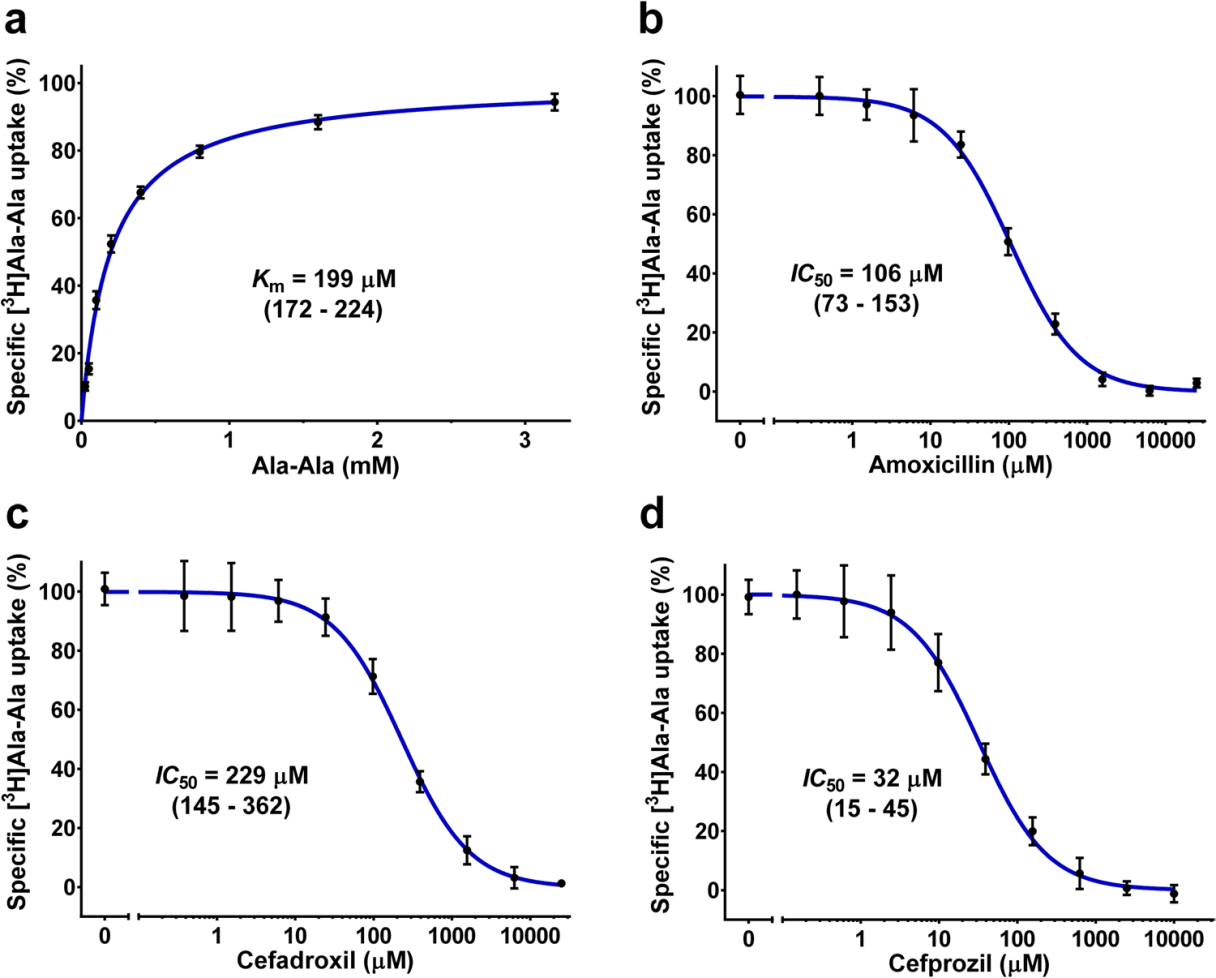
*K*_m_ and *IC*_50_ determinations for YePEPT^K314A-F311Y^. (**a**) Kinetics of [^3^H]Ala-Ala uptake in *E. coli* cells transformed with YePEPT^K314A-F311Y^. *IC*_50_ determination of YePEPT^K314A-F311Y^ for (**b**) amoxicillin, (**c**) cefadroxil and (**d**) cefprozil by heterologous competition. Data points in (**a**) represent vector-subtracted uptake of [^3^H]Ala-Ala normalized to *V*_max_ ± SEM of three experiments, each at least in triplicate. Data points in (**b**)–(**d**) represent vector-subtracted uptake of [^3^H]Ala-Ala normalized to the uninhibited signal ± SEM of three independent experiments, each at least in triplicate. Numbers in brackets below *K*_m_- and *IC*_50_-values represent 95% confidence intervals in µM.

Based on the results from the β-lactam antibiotic inhibition assay, YePEPT^K314A-F311Y^ represents a promising candidate for the desired antibiotic translocation module and was therefore functionally characterized in more detail. First, to investigate the kinetics of the uptake of the radioligand, the *K*_m_ for Ala-Ala was determined as 199 µM (Fig. 4a), which is the same as for YePEPT^WT^ ^32^. Next, the inhibition of [^3^H]Ala-Ala uptake by different concentrations of amoxicillin and cefadroxil, the two antibiotics showing the strongest inhibition in the initial screen, was evaluated, resulting in *IC*_50_-values of 106 µM (Fig. 4b) and 229 µM (Fig. 4c), respectively. While specificity for different β-lactam antibiotics differ between different members of the POT family, comparison of the obtained affinities for YePEPT^K314A-F311Y^ with the values for PEPT1 (*K*_i(amoxicillin)_ ≥ 10 mM, *K*_i(cefadroxil)_ = 7.2 mM)^22^ and PEPT2 (*K*_i(amoxicillin)_ = 430 µM, *K*_i(cefadroxil)_ = 3 µM)^23^ allow for the classification of YePEPT^K314A-F311Y^ as a high affinity transporter as is needed for an antibiotic transport module.

To deepen our understanding of the structure-affinity relationship of antibiotics in YePEPT^K314A-F311Y^ and to find antibiotics with higher affinities for YePEPT^K314A-F311Y^, we searched for commercially available analogues of amoxicillin and cefadroxil. Cefprozil (Fig. 2a 7), a cefadroxil analogue containing a longer aliphatic substituent on the six-membered ring of the cephem core structure, (i.e., a 1-propenyl- instead of a methyl group) showed a stronger inhibition than cefadroxil and amoxicillin with an *IC*_50_ of 32 µM (Fig. 4d).

### Evaluation of antibiotic transport by YePEPT^K314A-F311Y^

**Figure 5:**
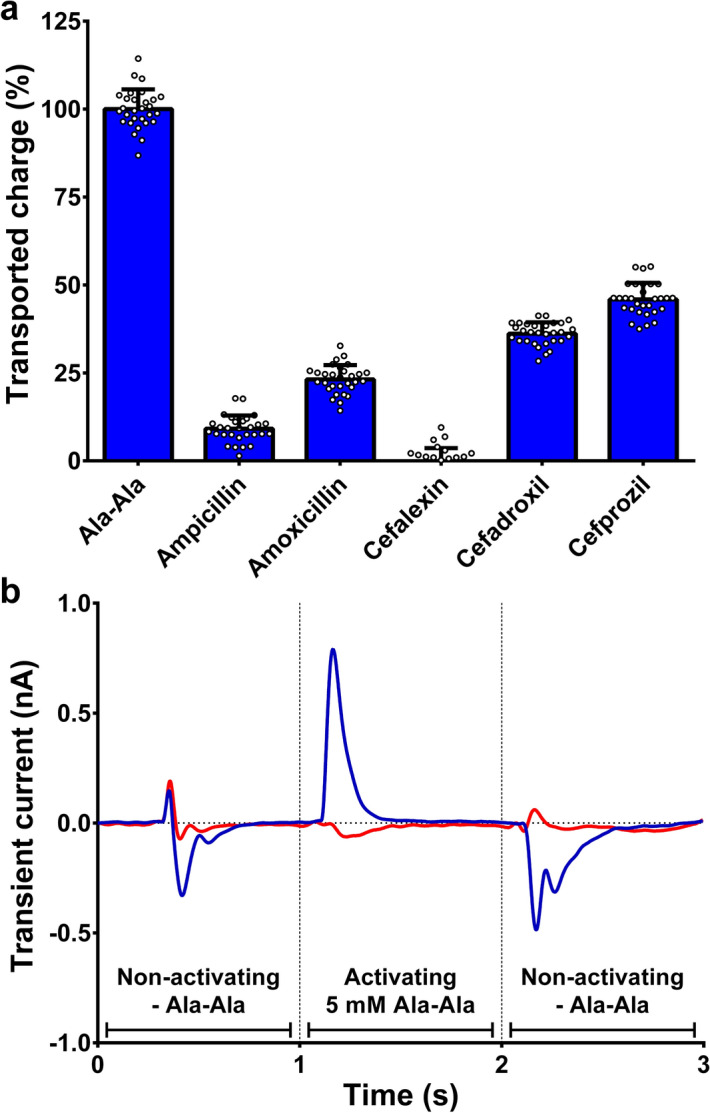
SSM-based electrophysiology of YePEPT^K314A-F311Y^. (**a**) Electrophysiology data of antibiotic transport by YePEPT^K314A-F311Y^. (**b**) Representative electrophysiology traces of Ala-Ala transport by proteoliposomes containing YePEPT^K314A-F311Y^ (blue) or empty control liposomes (red). Bars in (**a**) represent baseline-corrected transport normalized to the Ala-Ala signal ± SD from six sensors, each measured in quintuplicates. Individual measurements are depicted as white circles. Concentration of all compounds was 5 mM.

Considering that inhibition of Ala-Ala uptake does not provide proof that compounds are actually transported, we set up a solid-supported membrane (SSM)-based electrophysiology assay using proteoliposomes containing purified YePEPT^K314A-F311Y^ to evaluate transport of the antibiotics, which showed inhibition in the cell-based uptake (Fig. 5a). In short, during the assay, the proteoliposomes adsorbed to a SSM-chip are alternatingly perfused with non-activating- (i.e., without potential substrates) and activating solutions (i.e., containing the potential substrate to be evaluated). If electrogenic transport occurs, transient currents after solution exchange can be detected (Fig. 5b, in blue). As even small differences in solute concentrations (e.g., with or without the tested compound) can lead to artefact peaks, proteoliposome measurements were corrected by measuring the same solutions in liposomes devoid of transport protein (Fig. 5b, in red). This SSM-based electrophysiology technique for transporter research was described previously in detail^39,40^.

In all cases where transport occurred, a positive peak (i.e., net positive charge transport) was detected, indicating proton-coupled symport as expected for a POT-family member. Studies on the bacterial POT PepT_st_ showed proton:substrate stoichiometries of 3 for tripeptides and 4–5 for dipeptides^12,41^. Therefore and considering a partial negative charge (-0.23) of the tested antibiotics at pH 6.7 (Supplementary Table S1), the observed positive transport peaks from antibiotics (Supplementary Figure S2) are in line with the co-transport of at least one proton per antibiotic molecule. Both tested (amino-)penicillin antibiotics (i.e., ampicillin and amoxicillin) were transported with transported charges relative to Ala-Ala of 9% and 23%, respectively. The preference of YePEPT^K314A-F311Y^ for amoxicillin over ampicillin is in agreement with the higher specificity for antibiotics containing a hydroxyphenyl group observed in transport inhibition experiments (Fig. 2d). Of the (amino-)cephalosporins (i.e., cefalexin, cefadroxil and cefprozil) only the latter two were transported with relative transported charges of 36% and 46%, respectively. Cefadroxil being more efficiently transported than amoxicillin, despite the lower affinity (i.e., higher *IC*_50_-value) determined in heterologous competition experiments (Fig. 4b,c) suggests that YePEPT^K314A-F311Y^ preferentially transports β-lactam antibiotics containing a cephem ring structure (i.e., cephalosporins) over those containing a penam ring structure (i.e., penicillins). Nevertheless, in the case of cefalexin, the low specificity observed (Fig. 2d), due to the lack of a hydroxyl group at the phenyl ring, seems to outweigh this preference. The observed increased transport of cefprozil over cefadroxil can be attributed to the longer aliphatic side chain (Fig. 2a) either because of the higher affinity (Fig. 4c and d) or due to a structural preference of the transport mechanism of YePEPT^K314A-F311Y^.

## Conclusion

Vesicular molecular systems able to actively accumulate and degrade certain small organic molecules would be a promising approach to tackle the problem of environmental contamination by pollutants such as drugs and pesticides. One essential component needed for the development of such a system would be a transport module, which is able to accumulate the target molecule using energy provided by other components of the system (e.g., the proton gradient established by a light-driven proton pump). In this work, we engineered such a module specific for β-lactam antibiotics based on a bacterial peptide transporter. The specificity and affinity of the transporter YePEPT for certain β-lactam antibiotics was significantly improved using structure-based mutagenesis combined with functional characterization using an uptake assay in *E. coli* cells overexpressing the transporter. Two specific mutations were identified, which enhance the specificity and affinity of the transporter for β-lactam antibiotics containing certain structural features (i.e., hydroxyphenyl group or α-amino group). Screening of commercially available β-lactam antibiotics allowed for the identification of cefprozil as a ligand for YePEPT^K314A-F311Y^ with relatively high affinity (*IC*_50_ = 32 µM; Fig. 4d). Cefprozil represents a promising candidate for future co-crystallization studies with YePEPT^K314A-F311Y^ to elucidate the molecular binding mechanism for β-lactam antibiotics, which will facilitate a more targeted structure-based mutagenesis to further enhance the affinity for compounds of this group and to tailor the protein for compounds with specific structural features. Finally, SSM-based electrophysiology measurements provided evidence that β-lactam antibiotics with sufficient affinity not only inhibit transport (by binding competition), but are indeed translocated by YePEPT^K314A-F311Y^, with cefprozil reaching 46% transport relative to Ala-Ala. The extent to which certain β-lactam antibiotics are transported appears to be determined both, by the affinity and by structural features, as shown by the preference for hydroxyphenyl-containing cephalosporins over hydroxyphenyl-containing penicillins.

## Materials and methods

### Cloning of YePEPT

The gene of the peptide transporter YePEPT from *Y. enterocolitica* (UniProt accession number: R9G739) was amplified and inserted into the pZUDF vector^42^ as previously described^32^. Mutants were prepared by site-directed mutagenesis using the QuikChange Lightning Multi Site-Directed Mutagenesis Kit (Agilent Technologies).

### Uptake assay with *E. coli* cells overexpressing YePEPT variants

80 ml Luria Bertani (LB) medium supplemented with 100 µg/ml ampicillin was inoculated with 1 ml of an overnight pre-culture of *E. coli* BL21(DE3) pLysS transformed with YePEPT^WT^, YePEPT^K314A^, YePEPT^K314A-F311Y^ or empty vector (control), and incubated at 37 °C and 180 rpm. Protein expression was induced at an OD_600_ of 0.7–0.8 with 300 µM isopropyl-β-D-thiogalactopyranoside (IPTG). After 3 h of induction, cells corresponding to 10 ml of an OD_600_ of 1.5 were harvested by centrifugation (5000 × *g*, 4 °C, 15 min) and resuspended in 1.5 ml uptake buffer (50 mM HEPES–NaOH, 150 mM NaCl, 5 mM glucose, pH 7.5), and kept on ice.

The final volume of the samples for the uptake assay was 50 µl and consisted of 20 µl of resuspended cells, 10 µl of 5 × substrate Master mix (250 µM Ala-Ala (50 µM final concentration) in uptake buffer spiked with [^3^H]Ala-Ala (Campro Scientific) to a specific activity of 0.1 Ci/mmol) and 20 µl of competitor in uptake buffer. For screening, dipeptide and antibiotic competitors were used at a final concentration of 2.5 mM and 5 mM, respectively. For *K*_m_-determination, various concentrations of Ala-Ala, spiked to a specific activity of 0.0125 Ci/mmol were used. For *IC*_50_-determinations, various concentrations of competitor (Fig. 4) were used.

To be able to measure the substrate transport in the linear regime, duration and temperature of the assay were optimized for YePEPT variants: 100 s at 18 °C for YePEPT^WT^, 60 s at 18 °C for YePEPT^K314A^ and 100 s at 25 °C for YePEPT^K314A-F311Y^, see Supplementary Fig. S1. Transport was stopped after the indicated time by addition of 450 µl of ice-cold stop buffer (50 mM HEPES–NaOH, 150 mM NaCl, 5 mM glucose, 2.5 mM Ala-Ala, pH 7.5) and the cells were pelleted by centrifugation (14,000 × *g*, room temperature, 2 min), washed once with 450 µl uptake buffer and pelleted again. Washed cells were resuspended in 50 µl 5% (w/v) SDS and transferred to white 96-well plates (OptiPlate, PerkinElmer, Waltham, MA, USA). After addition of 150 µl scintillation cocktail (MicroScint 40, PerkinElmer), the plates were measured with a scintillation counter (2 min per well, Packard TopCount, PerkinElmer).

Raw data was processed by baseline-subtraction of the empty vector samples and normalized to the uninhibited [^3^H]Ala-Ala signal. *K*_m_- and *IC*_50_-values were determined by nonlinear regression. All data processing and plotting was performed with the Prism GraphPad 6 software.

### Overexpression and membrane isolation

24 l of LB medium supplemented with 100 µg/ml ampicillin were inoculated 1:100 with an overnight culture of *E. coli* BL21(DE3) pLysS transformed with pZUDF21-rbs-YePEPT-K314A-F311Y-3C-His_10_ and incubated at 37 °C and 180 rpm in an incubator shaker (Multitron, Infors HT). At an OD_600_ of 0.6–0.7, heterologous protein overexpression was induced by the addition of 300 µM IPTG and incubation was continued for 4 h. Cells were harvested by centrifugation (10,000 × *g*, 5 min, 4 °C). The pellet was washed once with 2 l of membrane wash buffer (20 mM Tris–HCl, 500 mM NaCl, pH 8), centrifuged again (10,000 × *g*, 5 min, 4 °C), resuspended in 300 ml of lysis buffer (20 mM Tris–HCl, 50 mM NaCl, pH 8) and stored at −80 °C. Cells were thawed and lysed by sonication for 60 min (total ON time) in 5 s ON/3 s OFF pulses using a tip sonifier (Branson 450 Digital Sonifier) while cooled in an ice bath. Membranes were then harvested by ultracentrifugation (150,000 × *g*, 1 h, 4 °C), washed once with 240 ml of membrane wash buffer and homogenized using a glass tissue homogenizer. The last ultracentrifugation was repeated once, the pellet resuspended in 36 ml of purification buffer (20 mM Tris–HCl, 300 mM NaCl, pH 8) and the membranes were flash-frozen in liquid nitrogen and stored at -80 °C.

### Purification of YePEPT^K314A-F311Y^

YePEPT^K314A-F311Y^ membranes from 2 l of cell culture were solubilized in 7 ml of purification buffer supplemented with 2% (w/v) of n-decyl-β-D-maltopyranoside (DM, Glycon Biochemicals GmbH) for 1 h at 4 °C under gentle agitation. After ultracentrifugation (150,000 × *g*, 1 h, 4 °C), the supernatant was diluted 1:1 with washing buffer (20 mM Tris–HCl, 300 mM NaCl, 5 mM L-histidine, 0.2% (w/v) DM, pH 8), supplemented with 500 µl (bed-volume) pre-equilibrated Ni–NTA superflow resin (Qiagen) and incubated for 4 h at 4 °C under gentle agitation. The resin was transferred to a column (Promega Wizard Midicolumns) and washed with 15 ml of washing buffer and 3 ml of elution buffer (20 mM Tris–HCl, 150 mM NaCl, 0.2% (w/v) DM, pH 8). After addition of 400 µl of elution buffer supplemented with 400 mM imidazol and incubation for 30 min at 4 °C under gentle agitation, the protein was eluted by centrifugation (3,000 × *g*, 1 min, 4 °C). Imidazol was removed using a desalting column (Zeba spin desalting columns 7 k MWCO, Thermo Scientific) pre-equilibrated with elution buffer.

### Reconstitution

*E. coli* polar lipids (Avanti polar lipids, Inc.) dissolved in chloroform were evaporated under a gentle stream of nitrogen, dried under vacuum overnight and rehydrated with elution buffer to a final concentration of 5 mg/ml. The lipids were then solubilized in elution buffer supplemented with 2% DM at a final lipid concentration of 2 mg/ml and incubated for 1 h at room temperature under gentle agitation. Purified YePEPT^K314A-F311Y^ was mixed with the solubilized lipids at an lipid-to-protein (LPR) ratio of 5 (1 mg/ml lipids, 0.2 mg/ml protein) and incubated for 10 min at room temperature under gentle agitation. The lipid-protein mixture was then transferred to 40 µl dialysis buttons (custom-made) with a 100,000 Da cut-off cellulose acetate membrane (Harvard apparatus) and dialysed against elution buffer for 6 days at 18 °C with two buffer exchanges (after 24 h and after 3 days). As a control sample, liposomes devoid of protein were prepared according to the same protocol.

### SSM-based electrophysiology transport assay

SSM-based electrophysiology experiments were performed using a SURF^2^ER N1 instrument (Nanion Technologies) according to published protocols^39,40^. SSM were prepared as follows: 50 µl of thiol solution (0.5 mM 1-octadecanethiol in 100% isopropanol) was added into the sensor well and incubated for 3 h in a closed petri dish. After removal of the thiol solution, the sensors were washed five times with 100% isopropanol and five times with Milli-Q ultrapure water. The SSM was formed by application of 1.5 µl of lipid solution (7.5 µg/µl 1,2-diphytanoyl-sn-glycero-3-phosphocholine in 100% n-decane) directly onto the gold surface, followed immediately by addition of 50 µl of non-activating buffer (25 mM MES, 25 mM HEPES, 140 mM KCl, 2 mM MgCl_2_, pH 6.7). Proteoliposomes containing YePEPT^K314A-F311Y^, as well as empty control liposomes, were diluted 1:1 with non-activating buffer, sonicated for 30 s in a bath sonicator and 5 µl adsorbed to each sensor. Before measurements, sensors were centrifuged (3000 × *g*, 30 min, 20 °C) and tested for suitable capacitance and conductance values^40^.

All measurements were conducted at 20 °C. Each measurement consisted of alternating perfusions for 1 s with non-activating- and activating buffer (non-activating buffer supplemented with 5 mM of the tested compound). Ala-Ala was measured at the beginning and at the end of each measurement sequence to test for possible signal loss of the sensor during the experiment. The transported charge of each measurement was determined by integrating the transient current peak occurring after perfusion with activating buffer. As even small differences in solute concentrations (e.g., with or without the tested compound) can lead to artefact peaks, the signals from sample sensors (sensors containing YePEPT^K314A-F311Y^-proteoliposomes) were corrected by subtracting the averaged signals from five control sensors (sensors with adsorbed liposomes devoid of protein), each measured in quintuplicates. Baseline-corrected signals from six sample sensors, each measured in quintuplicates, were then normalized to the Ala-Ala signal and merged. Measurements (including peak integration) were performed using the SURF^2^ER N1 control software. Data processing and plotting was performed with the Prism GraphPad 6 software.

## Supplementary Information

Supplementary Information.

## Data Availability

The datasets generated during and/or analysed during the current study are available from the corresponding author on reasonable request.
