# Supplementary material for: Engineering and functional characterization of a proton-driven β-lactam antibiotic translocation module for bionanotechnological applications
Source: Sci Rep. 2021 Aug 26;11:17205. doi: 10.1038/s41598-021-96298-4 (PMC8390754; doi:10.1038/s41598-021-96298-4)
Supplement: Supplementary file 1 — Supplementary Information. [file 41598_2021_96298_MOESM1_ESM.pdf]

Supplementary Information for:

Engineering and functional characterization of a proton-driven β-lactam antibiotic translocation module for bionanotechnological applications

Mirko Stauffer, Zöhre Ucurum, Daniel Harder & Dimitrios Fotiadis*

Institute of Biochemistry and Molecular Medicine, University of Bern, 3012 Bern, Switzerland

* Correspondence and requests for materials should be addressed to D.F. (email: dimitrios.fotiadis@ibmm.unibe.ch)

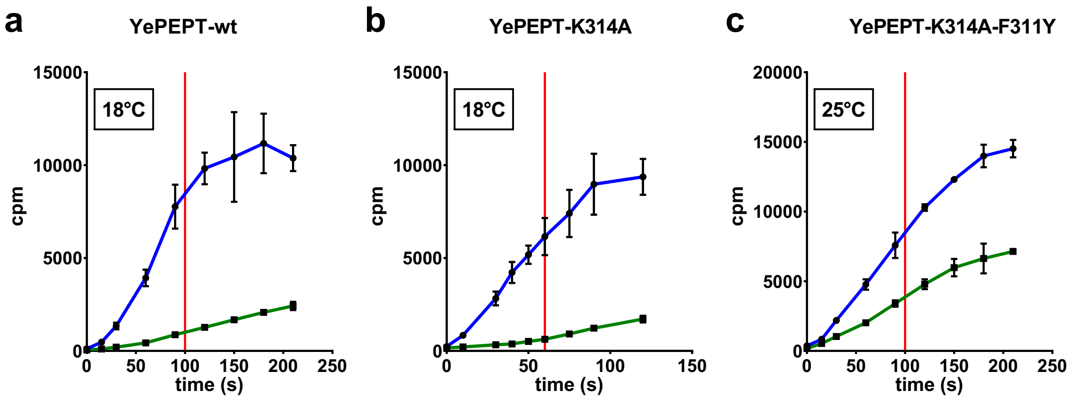

Supplementary Fig. S1 Representative time course analysis of [^{3}H]Ala-Ala uptake in *E. coli* cells transformed with different YePEPT variants (blue) or empty vector (green). Total signal of [^{3}H]Ala-Ala uptake by (**a**) YePEPT^{WT}, (**b**) YePEPT^{K314A} and (**c**) YePEPT$^{K314A\text{-}F311Y}$ at different time points and the indicated temperatures. Data points represent counts per minute (cpm) ± SEM of at least triplicates. The selected duration for uptake experiments of each YePEPT variant are indicated by a red, vertical line.

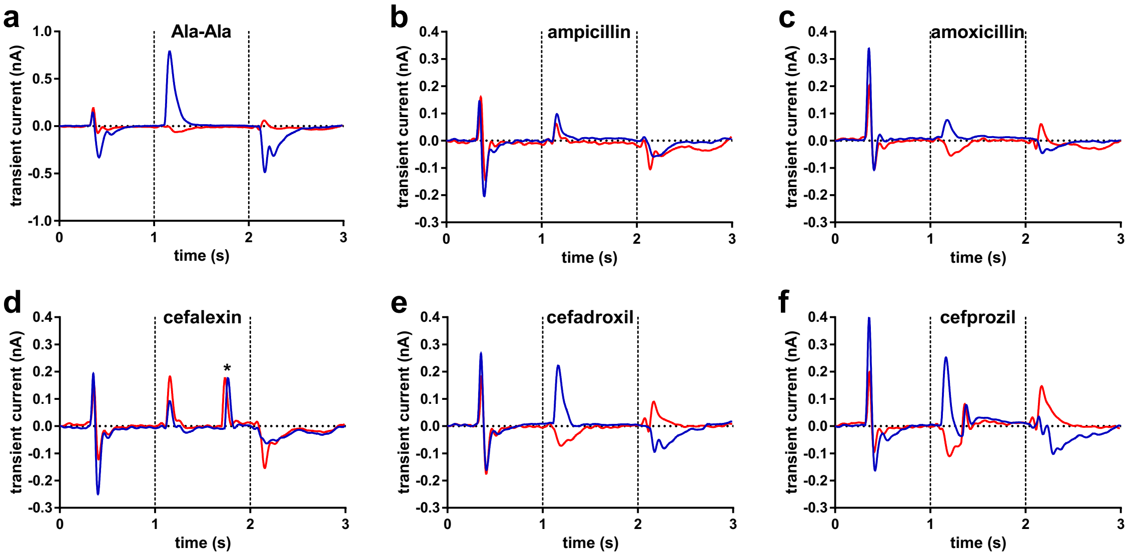

Supplementary Fig. S2 Representative electrophysiology traces for **a** Ala-Ala, **b** ampicillin, **c** amoxicillin, **d** cefalexin, **e** cefadroxil and **f** cefprozil of proteoliposomes containing YePEPT$^{K314A\text{-}F311Y}$ (blue) or empty control liposomes (red). In **d** a second peak (*) appeared consistently in all measurements, including when using control liposomes. Concentration of all compounds was 5 mM.

Supplementary Table S1 Isoelectric points (pI) and net charges of compounds used in the SSM-based electrophysiology transport assay.

Compound	Isoelectric point (pI)	Net charge at pH = 6.7
Ala-Ala	6.21	-0.02
Ampicillin	5.38	-0.23
Amoxicillin	5.38	-0.23
Cefalexin	5.49	-0.23
Cefadroxil	5.48	-0.23
Cefprozil	5.52	-0.23

All values were calculated using the Isoelectric Point plugin of the MarvinView software (ChemAxon Ltd.).
